# Global prevalence and types of complementary and alternative medicines use amongst adults with diabetes: systematic review and meta-analysis

**DOI:** 10.1007/s00228-021-03097-x

**Published:** 2021-03-08

**Authors:** Abdulaziz S. Alzahrani, Malcolm J. Price, Sheila M. Greenfield, Vibhu Paudyal

**Affiliations:** 1 School of Pharmacy, College of Medical and Dental Sciences, Sir Robert Aitken Institute for Medical Research, University of Birmingham, Birmingham, UK; 2 Institute of Applied Health Research, University of Birmingham, Birmingham, UK; 3 NIHR Birmingham Biomedical Research Centre, University Hospitals Birmingham NHS Foundation Trust and University of Birmingham, Birmingham, UK; 4 University Hospitals Birmingham NHS Foundation Trust, Birmingham, UK

**Keywords:** Prevalence, Complementary and alternative medicine, Diabetes, Systematic review

## Abstract

**Aim:**

This study aimed to undertake a systematic review and meta-analysis of global prevalence and types of complementary and alternative medicine (CAM) use amongst adults with diabetes.

**Methods:**

Nine databases, including MEDLINE and EMBASE, were searched for studies published between 2009 and 2019 which included extractable data for CAM use in adult patients with diabetes. Study characteristics, types of CAM, and overall and subgroup prevalence data in relation to CAM use were extracted. Meta-analysis of aggregate level data on prevalence and prevalence ratios (PRs) was performed using a random effects model.

**Results:**

From the 38 studies included in the review, a total of 37 types of CAM and 223 types of herbs were identified. Pooled prevalence of CAM use was 51%. A wide variation in prevalence rates (predictive interval 8–93%) was observed. In the context of high heterogeneity, we found no evidence that CAM use was associated with gender, chronicity or type of diabetes. Approximately one third of patients did not disclose their use of CAM to healthcare professionals (95% PrI 25%, 97%). Herbal medicines, acupuncture, homoeopathy and spiritual healing were the common CAM types reported.

**Conclusions:**

A wide variation in prevalence of CAM use by patients with diabetes was identified. Healthcare professionals should be aware of their patients’ use of CAM to ensure treatment optimization, avoid herb–drug interactions and promote medication adherence in diabetes. Diabetic reviews and clinical guidelines should incorporate exploration of patient use of CAM as many patients do not proactively disclose the use of CAM to their healthcare professionals.

**Registration:**

The protocol for this study was registered with the Centre for Review and Dissemination (CRD). Protocol registration number CRD42019125036.

**Supplementary Information:**

The online version contains supplementary material available at 10.1007/s00228-021-03097-x.

## Introduction

The World Health Organization (WHO) estimates that over 400 million people have diabetes worldwide, and this is projected to increase to reach 592 million by 2035 [1]. Poorly managed diabetes can lead to serious and possibly fatal complications such as cardiovascular disease, renal failure, nerve damage and blindness [2–4].

Diabetes mellitus (DM) is a chronic metabolic disorder in which blood glucose levels are higher than normal for a long period of time. These high blood glucose levels are attributed to abnormal disturbances of insulin production and/or function [5]. Diabetes is caused by either lack of insulin production by the pancreas (type 1 diabetes, T1D), when the amount of insulin produced by the pancreas is insufficient to carry out all blood glucose regulation processes, or by decreased insulin sensitivity by the body cells (type 2 diabetes, T2D). Diabetes can also be caused by a combination of low insulin production as well as low insulin sensitivity or be due to hormonal dysregulation in pregnancy [5].

Self-care practices relevant to self-management of diabetes include adherence to prescribed treatment and clinical management plans, adopting a healthy lifestyle and having a balanced diet [6]. In addition, many patients also use complementary and alternative medicine (CAM) [7]. The WHO defines CAM as a ‘broad set of health care practices that are not part of that country’s own tradition or conventional medicine and are not fully integrated into the dominant health-care system’ [8].

CAM use is known to be prevalent in patients with diabetes as a supplement to their existing orthodox diabetes treatments, as a replacement, or for reasons that might not be directly related to diabetes such as using CAM for energy and general wellbeing [7]. Various factors may influence CAM use by patients with diabetes. A study of 3978 U.S. adults suggested that CAM use by patients who were diabetic for more than 10 years or patients who had a functional limitation caused by diabetes were more likely to use CAM compared to patients with less severe diabetes [9]. In addition, the study reported that 77% of patients who used CAM for the treatment of diabetes used CAM as a supplement to conventional treatment, while 23% used CAM as a replacement [9].

CAM users often perceive CAM to be an effective means of lowering blood glucose levels and treating side effects of prescribed diabetic medications [10–15]. However, adverse outcomes of CAM use have also been reported. For example, CAM can affect the management of diabetes by either direct herb–drug interaction with the use of herbal remedies or indirectly by affecting medication adherence when using herbal or any other CAM types [6].

There is a lack of an up-to-date systematic review that investigates the prevalence of CAM use by patients with diabetes. Patient sources of health-related information have changed immensely in the past decade [16]. In particular, increasing availability and use of web-based information sources, including social media and online health information in recent years, may encourage and inhibit CAM use in long term health conditions [17]. An up-to date systematic review on the prevalence of CAM use by patients with diabetes will help healthcare professionals to consider patient use of CAM when counselling patients, supporting adherence and identifying the risks of interactions and adverse effects when CAMs are used in conjunction with prescribed treatments.

The aim of this study was to systematically review the global prevalence of CAM use amongst adults with diabetes. Specific objectives were to identify the types of CAM that are used by the population with diabetes and to identify differences in CAM use amongst different populations with diabetes, including types of diabetes, demographic characteristics, duration of diabetes and presence or absence of diabetic complications.

### Methods

This systematic review was informed by the Preferred Reporting Items for Systematic Reviews and Meta-Analyses (PRISMA) guidelines and checklist [18]. A protocol was developed as per the PRISMA protocol guideline (protocol ID CRD42019125036).

#### Data sources and searches

Cochrane Library, MEDLINE, Embase, CINAHL, AMED, Web of Science, Google Scholar and PROSPERO databases were searched for the past 10 years covering 2009 to June 2019. Open Grey was searched for grey literature. Search terms and an example search strategy are listed in Supplementary Table 1. The review was restricted to studies published in English. Studies that recruited participants who are adult patients with diabetes, 18 years of age and older and reported partially or exclusively the prevalence and use of CAM amongst patients with diabetes were included. Studies which either focused on CAM use in conjunction with conventional treatments or as a replacement were considered.

#### Study selection

Screening and selection were performed independently by two review authors (AA, VP) and were carried out in three phases. Titles and abstracts were screened for inclusion of possible relevant studies followed by assessment of full texts for eligibility. Reference lists of included studies were screened. If a title was considered relevant; the study was manually searched and the abstract examined.

#### Data extraction and quality assessment

Data on study characteristics, prevalence of CAM use as well as types of CAM used by patients with diabetes were extracted. Two review authors (AA, VP) independently assessed the quality of included studies using the critical appraisal tool from the Joanna Briggs Institute (JBI) checklist [19]. Studies were classified into high, moderate and low quality based on the results of the JBI checklist (Supplementary Table 1). The quality assessment in included studies was focused on three fields: clarity of inclusion criteria and study setting and sampling, appropriateness of approaches to data collection and analysis, and outcome measurement (i.e. use of CAM). Included studies were judged to be of ‘high quality’ if quality criteria were satisfied by at least 7 items, ‘moderate quality’ for scores of 3–6 and ‘low quality’ for scores ≤2 [20]. All studies were included regardless of their quality.

#### Data synthesis and analysis

A quantitative synthesis of aggregate level data on prevalence was performed. Study specific results were reported as percentage prevalence with exact 95% confidence intervals (95%CI). When sufficient data were available for within-study comparisons of prevalence between dichotomous groups, e.g. sex, then relative prevalence ratios (PRs) together with 95% confidence intervals were calculated. Meta-analyses of proportions and PRs were performed using a random effects model fit using the method of Der Simonian & Laird [21]. Heterogeneity was assessed using the I^2^ statistic, the between study standard deviation and calculation of 95% prediction intervals (95%PrI) for the prevalence in a new study [22, 23]. Data are presented in forest plots which include pooled estimates where appropriate. All analyses were performed using STATA version 15.

## Results

**Fig. 1:**
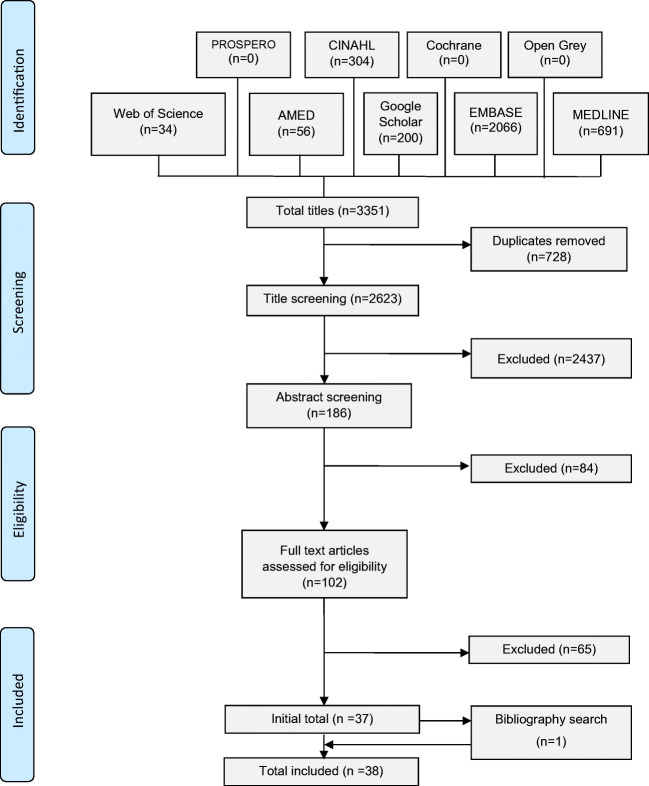
Prisma flow diagram

A total of 2623 unique titles were screened of which 38 articles met the inclusion criteria (Fig. 1). After applying quality assessment, studies fell into these categories (8 high quality studies, 30 moderate quality studies and no low quality studies). Details of critical appraisal results are available in Supplementary Table 2.

### Study characteristics

Included studies originated from 25 different countries. Participants were mostly recruited from diabetes clinics and healthcare centres (Table 1). Fifteen of the studies enrolled participants with either T1D or T2D, and 23 studies only included patients with T2D. Out of the included 38 studies, 37 were cross-sectional surveys and one analysed data from another cohort study (Table 1).

**Table 1:** Study characteristics

Author and year	Country of study	Focus of the study	Study settings and recruitment of participants	Study design	Data collection method	Study participants
Alami et al. 2015 [24]	Morocco	Herbal supplements only	Mohammad VI university hospital,Oujda	Cross-sectional	Face-to-face interview using a semi-structured questionnaire	T1D and T2D patients
Al-Eidi et al. 2016 [25]	Saudi	Any CAM type	Diabetic Centre of King Salman bin Abdul-Aziz Hospital, in Riyadh city	Cross-sectional	Face-to-face interview using a structured questionnaire	T2D patients
Al-garni et al. 2017 [26]	Saudi	Herbal supplements only	Jeddah Diabetic Centre (JDC)	Cross-sectional	Interviewer-administered semi-structured questionnaire	T2D patients
Ali-Shtayehet et al. 2012 [14]	Palestine	Any CAM type	Patients attending outpatient departments at Governmental Hospitals in 7 towns in the Palestinian territories (Jenin, Nablus, Tulkarm, Qalqilia, Tubas, Ramalla, and Hebron)	Cross-sectional	Structured questionnaires	T1D and T2D patients
Amaeze et al. 2018 [27]	Nigeria	Herbal supplements only	5 secondary healthcare facilities across Lagos State	Cross-sectional	Interviewer-administered questionnaires	T2D patients
Andrews et al. 2018 [28]	Guatemala	Any CAM type	Interview three groups in the San Lucas Tolimán area	Cross-sectional	Semi-structured questionnaires	T2D patientsHealth promotersTraditional healers
Ashur et al. 2017 [29]	Libya	Any CAM type	National Centre for Diabetes and Endocrinology in Tripoli	Cross-sectional	Self-administered structured questionnaire	T2D patients
Avci et al. 2018 [30]	Turkey	Any CAM type	Van Yuzuncu Yil University, Van	Cross-sectional	Semi-structured questionnaires	T1D and T2D patients
Azizi-Fini et al. 2016 [11]	Iran	Herbal supplements only	Golabchi and Naqavi diabetes clinics in the Kashan city	Cross-sectional	Interviewer-administered structured questionnaires	T2D patients
Baharom et al. 2016 [31]	Malaysia	Any CAM type	45 government health clinics across Nigeria Sembilan	Cross-sectional	Interviewer-administered structured questionnaires	T2D patients
Bradley et al. 2012 [13]	USA	Any CAM type	Patients with moderately to poorly controlled T2D who receive care from Group Health Cooperative (GHC), a large non-profit, integrated health care system in Washington State	Cross-sectional	Telephone-administered questionnaires.	T2D patients
Candar et al. 2018 [32]	Turkey	Any CAM type	Patients registered with the Bursa Yuksek Ihtisas Training and Research Hospital Education Family Health Centre	Cross-sectional	Questionnaires	T1D and T2D patients
Chao et al. 2014 [33]	USA	Any CAM type	Patients who received primary care at one of four publicly funded clinics in the Community Health Network of San Francisco	Cross-sectional	Data collected for the Self-Management Automated and Real-Time Telephonic Support (SMART Steps) Study	T2D patients
Ching et al. 2013 [34]	Malaysia	Any CAM type	Primary health care clinic at Salak in Sepang	Cross-sectional	Face-to-face interview using a structured questionnaire	T2D patients
Damnjanovic et al. 2015 [35]	Serbia	Herbal supplements only	6 Remedia Pharmacy HealthFacilities in the territory of Nis	Cross-sectional	Structured questionnaires	T2D patients
Devi et al. 2015 [36]	India	Any CAM type	Diabetes Health camp conducted by VS micro lab, Madurai	Cross-sectional	Structured questionnaires	T2D patients
Fabian et al. 2011 [37]	Austria	Herbal supplements only	Diabetes Centre of the Division of Endocrinology and Metabolism, Department of Internal Medicine, Medical University of Graz	Cross-sectional	Face-to-face interview using a structured questionnaire	T1D and T2D patients
Fan et al. 2013 [38]	Singapore	Any CAM type	Single centre study conducted in an outpatient diabetes Centre with an average load of 2500 patients a month	Cross-sectional	Self-administered questionnaires.	T2D patients
Hashempur et al. 2015 [39]	Iran	Any CAM type	Two outpatient diabetes clinics affiliated with the Shiraz University of Medical Sciences, Shiraz	Cross-sectional	Face-to-face interview using semi-structured questionnaire	T1D and T2D patients
Kamel et al. 2017 [40]	Saudi	Herbal supplements only	King Abdul-Aziz University and King Fahad General Hospitals in Jeddah	Cross-sectional	Interviewer-administered structured questionnaires	T1D and T2D patients
Karaman et al. 2018 [10]	Turkey	Herbal supplements only	Endocrinology clinics of two hospitals in Izmir	Cross-sectional	Face-to-face interview using a structured questionnaire	T1D and T2D patients
Khalaf and Whitford 2010 [41]	Bahrain	Any CAM type	Patients attending two hospital diabetes clinics	Cross-sectional	Questionnaires (administration not detailed)	T1D and T2D patients
Khalil et al. 2013 [42]	Egypt	Herbal supplements only	Outpatient clinics of Alexandria University Hospital, from seven health insurance centres, six MOH hospitals, and one private healthcare facility.	Cross-sectional	Questionnaires (administration method not reported)	T2D patients
Koren et al. 2015 [43]	Israel	Herbal supplements only	Internal medicine department at Assaf Harofeh Medical Centre, Zerifin	Cross-sectional	Interviewer-administered structured questionnaires	T2D patients
Lui et al. 2012 [44]	Australia	Any CAM type	Data reported here are taken from the Living with Diabetes Study (LWDS), a five-year, prospective cohort study being conducted in the State of Queensland	Data from cohort study	Questionnaires (administration not detailed)	T1D and T2D patients
Lunyera et al. 2016 [45]	Tanzania	Herbal supplements only	Kilimanjaro Region of Tanzania	Cross-sectional	Verbally administered structured questionnaire	T1D and T2D patients
Medagama et al. 2014 [46]	Sri Lanka	Herbal supplements only	Diabetes clinic at Teaching Hospital Peradeniya	Cross-sectional	Face-to-face interview using a structured questionnaire	T2D patients
Mekuria et al. 2018 [47]	Ethiopia	Herbal supplements only	Diabetes care clinic of University of Gondar comprehensive specialized hospital	Cross-sectional	Interviewer-administered questionnaires	T2D patients
Mohamed Ali and Mahfouz 2014 [48]	Sudan	Herbal supplements only	125 primary health care centres in Khartoum	Cross-sectional	Interviewer-administered questionnaires	T2D patients
Naja et al. 2014 [49]	Lebanon	Any CAM type	Patients recruited from two major referral centres in Beirut- a public hospital and a private academic medical Centre	Cross-sectional	Face-to-face interview using a structured questionnaire	T2D patients
Nguyen et al. 2014 [50]	USA	Any CAM type	Patients recruited from seven primary care or endocrinology clinics affiliated with an academic medical centre in Southern California	Cross-sectional	Self-administered structured questionnaire	T2D patients
Putthapiban et al. 2017 [51]	Thailand	Herbal supplements only	At the Endocrine Clinic in Ramathibodi Hospital, Bangkok	Cross-sectional	Face-to-face interview using a structured questionnaire	T2D patients
Rhee et al. 2018 [52]	USA	Any CAM type	Non-institutionalized civilians in US	Cross-sectional	Data were from the 2012 NHIS, which was administrated by the National Centre for Health Statistics of the Centers for Disease Control and Prevention	T1D and T2D patients
Sethi et al. 2011 [12]	India	Any CAM type	Tertiary care Centre in Delhi	Cross-sectional	Face-to-face interview using a Semi-structured questionnaire	T1D and T2D patients
Vishnu et al. 2017 [53]	India	Any CAM type	Rural Kollam district of the Indian state of Kerala (community based)	Cross-sectional	Interviewer-administered structured questionnaires	T1D and T2D patients
Wanchai and Phrompayak 2016 [54]	Thailand	Any CAM type	Four primary healthcare unitsand two secondary hospitals in the north of Thailand	Cross-sectional	Semi-structured questionnaire	T2D patients
Wazaify et al. 2011 [15]	Jordan	Herbal supplements only	Outpatient departments at The National Centre for Diabetes, Endocrine and Genetics (NCDEG.	Cross-sectional	Face-to-face interview using a Semi-structured questionnaire	T1D and T2D patients
Yildirim and Marakoglu 2018 [55]	Turkey	Any CAM type	Outpatient diabetes from Selçuk University Family Medicine Diabetes Education Clinic	Cross-sectional	Face-to-face interview using a structured questionnaire	T2D patients

*CAM* Complementary and alternative medicine

### Types of CAM

Sixteen studies focused exclusively on herbal and nutritional supplement use by patients with diabetes (Table 1). The remaining 22 studies discussed other CAM types. Fourteen of those 22 studies that investigated other CAM types also reported the use of herbal and nutritional supplements as a form of CAM [12–14, 25, 32, 34, 36, 38, 39, 41, 49, 52–54]. A total of 35 different CAM types were reported in at least one study. CAM types used by patients with diabetes and mentioned in the most studies were acupuncture (*n* = 6 studies), Mind–body therapies (n = 6 studies) religious and spiritual healing (*n* = 5 studies) and homoeopathy (*n* = 4 studies) (Table 2).

**Table 2:** List of complementary and alternative medicine types as cited by included studies

CAM forms (other than herbal supplements)	Studies cited the CAM form	CAM forms (other than herbal supplements)	Studies cited the CAM form
Acupuncture	[25, 29, 36, 39, 50, 52]	Ruqyah (recitation) with the Quran	[25, 29]
Mind–body therapies	[32, 34, 36, 39, 41, 52]	Ruqyah water or oil	[25, 29]
Religious and spiritual healing	[29, 32, 49, 50, 54]	Balneotherapy	[32]
Homoeopathy	[12, 36, 52, 53]	Biofeedback	[52]
Meditation	[13, 36, 52, 54]	Chelation	[52]
Massage	[13, 25, 38, 49]	Chinese medicine	[49]
Ayurveda	[36, 52, 53]	Curandero	[50]
Chiropractic Massage	[13, 50, 52]	Daode Xinxi	[54]
Energy therapies	[34, 41, 52]	Deep breathing exercises	[13]
Specific diet	[13, 25, 36]	Leech (Hirudotherapy)	[32]
Yoga	[13, 36, 52]	Music therapy	[36]
Al-hijama (wet cupping)	[25, 29]	Prayer by religion person (imam)	[30]
Biologically based therapies	[36, 52]	Progressive muscle relaxation	[13]
Cupping	[32, 39]	Qi gong	[52]
Folk medicine	[13, 49]	Sugar therapy	[53]
Honey	[14, 25]	Tai chi	[52]
Movement therapies	[36, 52]	Traditional healers	[52]
Naturopathy	[50, 52]		

*CAM* Complementary and alternative medicine

Within the 31 studies which reported the use of herbal and nutritional supplements by patients with diabetes, a total of 223 different herbal and nutritional supplements were reported (Supplementary Table 3). The five herbs that were mentioned in the most studies were, cinnamon (*Cinnamomum verum*) and fenugreek (*Trigonella foenum-graecum*) each reported in 18 different studies, garlic (*Allium sativum*) reported in 17 studies, *aloe vera* (*Aloe Vera*) reported in 14 studies and black seed (*Nigella sativa*) reported in 12 studies.

### Prevalence of CAM use

**Fig. 2:**
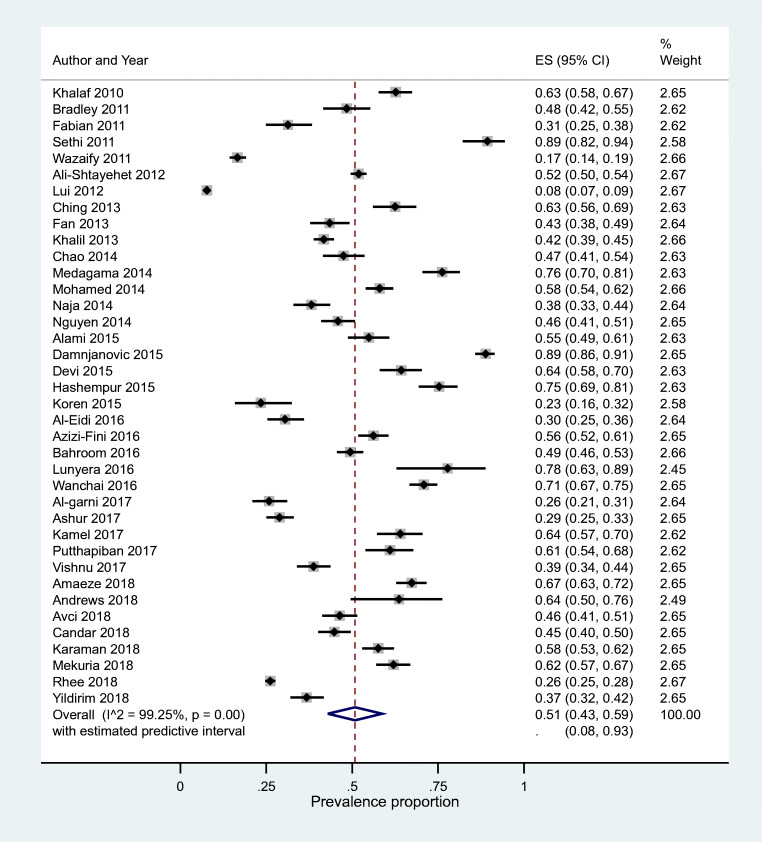
Forest plot showing pooled prevalence of complementary and alternative medicine in diabetes

The highest prevalence of CAM (all types) use was reported at 89% by two studies, one each from India and Jordan followed by studies in Tanzania (78%), Sri Lanka (76%) and Iran (75%) (Table 3) [12, 35, 39, 45, 46]. The lowest prevalence of CAM use was 17% as reported by a study conducted in Jordan [15]. A study in Australia reported a prevalence of 8%, but the study gathered data from patients about their visits to CAM practitioners only and did not include data on CAM use in general by patients with diabetes [44]. Other studies reporting the lowest prevalence of CAM use included studies in Libya (29%), Saudi Arabia (26%), USA (26%), Israel (23%) and Jordan (17%) [15, 26, 29, 43, 52]. Pooled prevalence of CAM use was 51% (95%CI 43%, 59%). However, heterogeneity was very high (I^2^ = 99%) with the predictive interval ranging from 8% to 93%. (Fig. 2).

**Table 3:** Included studies’ overall and subgroups prevalence of CAM use

Country	Study	Sample size	Prevalence of CAM use	All participants		Female			Male			T1D			T2D			Had diabetes for ≤5y			Had diabetes for >5y		
				users	non users	users	non users	%	users	non users	%	users	non users	%	users	non users	%	users	non users	%	users	non users	%
India	Sethi et al. 2011 [12]	113	89.38%	101	12	NR	NR	NR	NR	NR	NR	NR	NR	NR	NR	NR	NR	NR	NR	NR	NR	NR	NR
Serbia	Damnjanovic et al. 2015 [35]	519	88.82%	461	58	261	15	95%	200	43	82%	NR	NR	NR	NR	NR	NR	NR	NR	NR	NR	NR	NR
Tanzania	Lunyera et al. 2016 [45]	45	77.78%	35	10	NR	NR	NR	NR	NR	NR	NR	NR	NR	NR	NR	NR	NR	NR	NR	NR	NR	NR
Sri Lanka	Medagama et al. 2014 [46]	252	76.19%	192	60	139	28	83%	53	32	62%	NR	NR	NR	NR	NR	NR	NR	NR	NR	NR	NR	NR
Iran	Hashempur et al. 2015 [39]	239	75.31%	180	59	124	37	77%	56	22	72%	10	7	59%	170	52	77%	80	25	76%	100	34	75%
Thailand	Wanchai and Phrompayak 2016 [54]	508	70.87%	360	148	282	102	73%	78	46	63%	NR	NR	NR	NR	NR	NR	NR	NR	NR	NR	NR	NR
Nigeria	Amaeze et al. 2018 [27]	453	67.33%	305	148	98	45	69%	207	103	67%	NR	NR	NR	NR	NR	NR	96	66	59%	209	82	72%
India	Devi et al. 2015 [36]	252	64.29%	162	90	98	41	71%	64	49	57%	NR	NR	NR	NR	NR	NR	55	40	58%	107	50	68%
Saudi	Kamel et al. 2017 [40]	214	64.02%	137	77	84	44	66%	53	33	62%	50	20	71%	87	57	60%	NR	NR	NR	NR	NR	NR
Guatemala	Andrews et al. 2018 [28]	55	63.64%	35	20	NR	NR	NR	NR	NR	NR	NR	NR	NR	NR	NR	NR	NR	NR	NR	NR	NR	NR
Bahrain	Khalaf and Whitford 2010 [41]	402	62.69%	252	150	149	69	68%	103	81	56%	NR	NR	NR	NR	NR	NR	48	51	48%	204	99	67%
Malaysia	Ching et al. 2013 [34]	240	62.50%	150	90	96	49	66%	54	41	57%	NR	NR	NR	NR	NR	NR	NR	NR	NR	NR	NR	NR
Ethiopia	Mekuria et al. 2018 [47]	387	62.02%	240	147	149	73	67%	91	74	55%	NR	NR	NR	NR	NR	NR	168	60	74%	72	87	45%
Thailand	Putthapiban et al. 2017 [51]	200	61.00%	122	78	76	42	64%	46	36	56%	NR	NR	NR	NR	NR	NR	NR	NR	NR	NR	NR	NR
Sudan	Mohamed Ali and Mahfouz 2014 [48]	600	58.00%	348	252	206	167	55%	142	85	63%	NR	NR	NR	NR	NR	NR	67	76	47%	281	176	61%
Turkey	Karaman et al. 2018 [10]	455	57.58%	262	193	225	148	60%	37	45	45%	53	49	52%	209	114	65%	51	62	45%	211	131	62%
Iran	Azizi-Fini et al. 2016 [11]	500	56.20%	281	219	203	153	57%	78	66	54%	NR	NR	NR	NR	NR	NR	NR	NR	NR	NR	NR	NR
Morocco	Alami et al. 2015 [24]	279	54.84%	153	126	117	83	59%	36	43	46%	36	43	46%	117	83	59%	NR	NR	NR	NR	NR	NR
Palestine	Ali-Shtayehet et al. 2012 [14]	1883	51.89%	977	906	519	470	52%	458	436	51%	114	84	58%	863	822	51%	341	325	51%	636	581	52%
Malaysia	Baharom et al. 2016 [31]	680	49.41%	336	344	224	175	56%	112	169	40%	NR	NR	NR	NR	NR	NR	NR	NR	NR	NR	NR	NR
USA	Bradley et al. 2012 [13]	219	48.40%	106	113	47	50	48%	59	63	48%	NR	NR	NR	NR	NR	NR	NR	NR	NR	NR	NR	NR
USA	Chao et al. 2014 [33]	278	47.48%	132	146	101	38	73%	31	108	22%	NR	NR	NR	NR	NR	NR	NR	NR	NR	NR	NR	NR
Turkey	Avci., 2018 [30]	386	46.37%	179	207	95	122	44%	84	85	50%	29	16	64%	150	191	44%	68	107	39%	111	100	53%
USA	Nguyen et al. 2014 [50]	410	45.85%	188	222	NR	NR	NR	NR	NR	NR	NR	NR	NR	NR	NR	NR	NR	NR	NR	NR	NR	NR
Turkey	Candar et al. 2018 [32]	442	44.80%	198	244	137	134	51%	61	110	36%	NR	NR	NR	NR	NR	NR	NR	NR	NR	NR	NR	NR
Singapore	Fan et al., 2013 [38]	304	43.42%	132	172	67	69	49%	65	103	39%	NR	NR	NR	NR	NR	NR	NR	NR	NR	NR	NR	NR
Egypt	Khalil et al. 2013 [42]	1100	41.73%	459	641	252	359	41%	207	282	42%	NR	NR	NR	NR	NR	NR	87	202	30%	372	439	46%
India	Vishnu et al. 2017 [53]	400	38.75%	155	245	73	109	40%	82	136	38%	NR	NR	NR	NR	NR	NR	95	142	40%	60	103	37%
Lebanon	Naja et al. 2014 [56]	333	38.14%	127	206	51	98	34%	76	108	41%	NR	NR	NR	NR	NR	NR	34	77	31%	93	129	42%
Turkey	Yildirim and Marakoglu 2018 [55]	400	36.75%	147	253	91	115	44%	56	138	29%	NR	NR	NR	NR	NR	NR	77	149	34%	70	104	40%
Austria	Fabian et al. 2011 [37]	198	31.31%	62	136	NR	NR	NR	NR	NR	NR	NR	NR	NR	NR	NR	NR	NR	NR	NR	NR	NR	NR
Saudi	Al-Eidi et al. 2016 [25]	302	30.46%	92	210	50	121	29%	42	89	32%	NR	NR	NR	NR	NR	NR	24	93	21%	68	117	37%
Libya	Ashur et al. 2017 [29]	523	28.87%	151	372	102	206	33%	49	166	23%	NR	NR	NR	NR	NR	NR	86	226	28%	65	146	31%
USA	Rhee et al. 2018 [52]	3386	26.17%	886	2500	NR	NR	NR	NR	NR	NR	NR	NR	NR	NR	NR	NR	NR	NR	NR	NR	NR	NR
Saudi	Al-garni et al. 2017 [26]	310	25.81%	80	230	NR	NR	NR	NR	NR	NR	NR	NR	NR	NR	NR	NR	NR	NR	NR	NR	NR	NR
Israel	Koren et al. 2015 [43]	111	23.42%	26	85	12	37	24%	14	48	23%	NR	NR	NR	NR	NR	NR	NR	NR	NR	NR	NR	NR
Jordan	Wazaify et al. 2011 [15]	1000	16.60%	166	834	99	432	19%	67	402	14%	8	44	15%	158	790	17%	NR	NR	NR	NR	NR	NR
Australia	Lui et al. 2012 [44]	3337	7.73%	258	3079	157	1727	8%	101	1352	7%	NR	NR	NR	NR	NR	NR	NR	NR	NR	NR	NR	NR

*CAM* Complementary and alternative medicine, *NR* Not reported

### Subgroup analysis

#### Study level factors

**Fig. 3:**
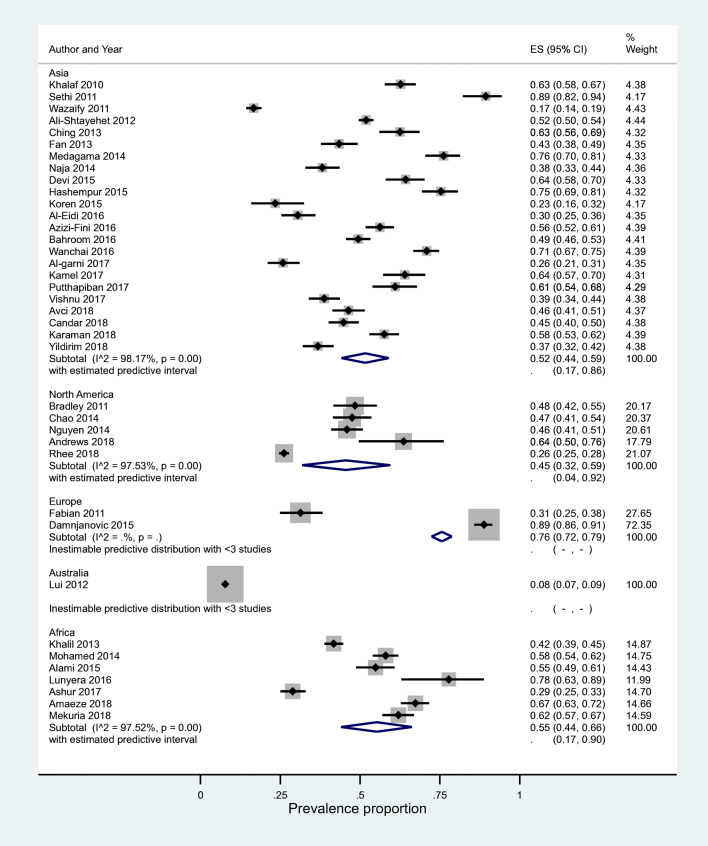
Meta-analysis of study level factors in relation to CAM use (prevalence proportion by continent). CAM, complementary and alternative medicine

Meta-analysis was conducted for results stratified at the study level by continent. I^2^ was 97.5% and predictive intervals were found to be wide. The highest prevalence rates of 76% were observed in Europe (PrI inestimable), followed by Africa 55%, (95%PrI 0.17, 0.90) from seven studies. The lowest prevalence rates were observed in North America 45%, (95%PrI 0.04, 0.92) from five studies (Fig. 3).

#### Patient level factors

**Fig. 4:**
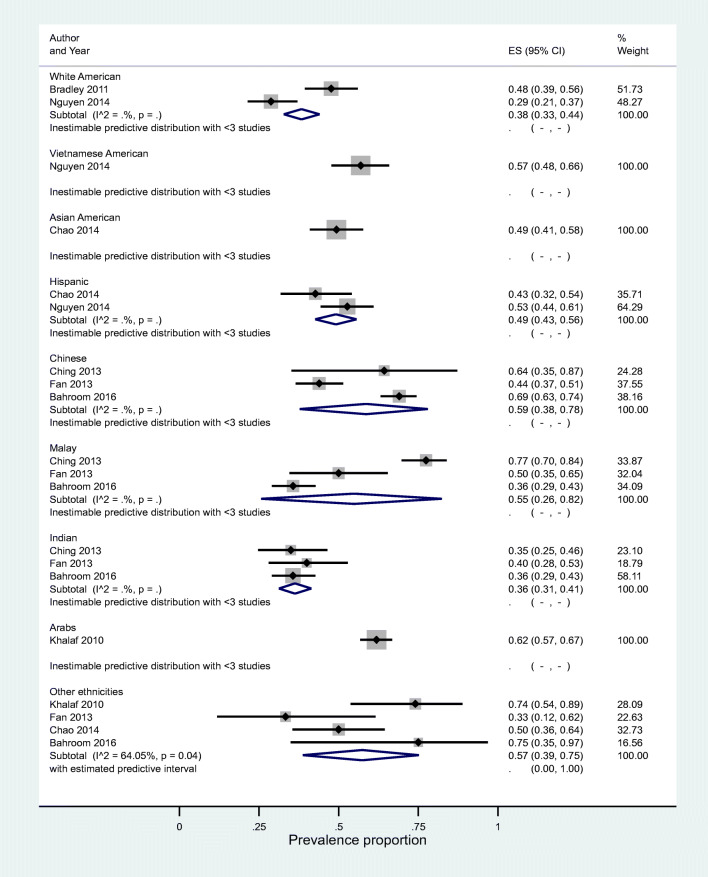
Ethnicity subgroup forest plot showing prevalence of CAM use. CAM, Complementary and alternative medicine

Subgroup analyses were conducted across ethnicity (reported in eight studies). All meta-analyses at subgroup level also showed high levels of heterogeneity. Results were as follows: for the ethnicity subgroup, no predictive interval could be estimated other than PrI for the group of ‘other ethnicities’ prevalence ratio 0.57 (95%CI 0.39–0.75); the estimated predictive intervals ranged between 0.00 and 1.00, I^2^ = 64.05%) (Fig. 4).

For analysis stratified by binary subgroups within-study comparative data were extractable for sex (31 studies), type of diabetes (7 studies), duration of diabetes (15 studies) and presence or absence of diabetic complications (10 studies). Within study pooled estimates PRs for patients with no diabetic complications versus patients with diabetic complications gave a prevalence ratio (PR) 0.81 (95%CI 0.66, 0.99), (95%PrI 0.39–1.67) (I^2^ = 89%) (Supplementary Fig. 1). For patients who had had diabetes for more than 5 years versus less than 5 years pooled PR was 1.71 (95%CI 1.04, 1.32), (95%PrI 0.73, 1.88) (I^2^ of 83%) (Supplementary Fig. 2). For male versus female participants, pooled PR was 0.86 (95%CI 0.81, 0.91), (95%PrI 0.64, 1.16) (I^2^ of 72%) (Supplementary Fig. 3). Pooled PR for patients with T2D versus T1D patients was 1.00 (95%CI 0.83, 1.20), 95%(PrI 0.56, 1.77) (I^2^ = 75%) (Supplementary Fig. 4).

#### Additional outcomes

##### CAM as a complementary or alternative treatment

Eight of the 38 included studies assessed whether CAM was used as an additional treatment or as an alternative treatment to conventional medicines. Prevalence of CAM use as an additional treatment to prescribed medicine was 78% (95%CI 56%, 94%) with 95% PrI (4%, 1.00%) (I^2^ = 98%), and the percentage of patients who used CAM as an alternative to their prescribed medicine was 21% (95%CI 12%, 31%) with 95% PrI (0.%, 63%) (I^2^ = 89%) (Supplementary Figs. 5, 6).

##### Patients’ disclosure of CAM use to healthcare professionals

**Fig. 5:**
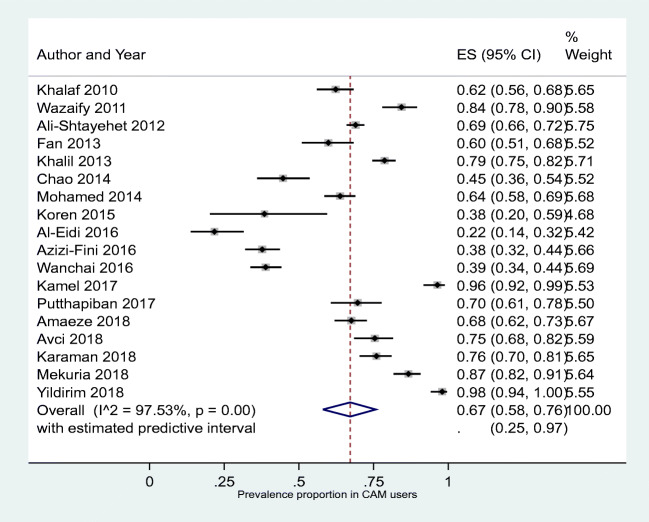
Patient who do not disclose the use of CAM to the healthcare professionals. CAM, complementary and alternative medicine

The percentage of patients who do not disclose their CAM use to healthcare professionals was 67% (95%CI 58%, 76%) with 95% PrI (25%, 97%) (I^2^ = 98%) (Fig. 5).

## Discussion

This study provides up-to-date data on the global prevalence of CAM use by patients with diabetes as reported in the peer reviewed research literature. The last literature review on CAM use of patients with diabetes was published in 2007 [7] which reviewed studies conducted in nine countries and reported prevalence ranging from 17% to 73%. A similarly wide variation in prevalence rate of 8–89% was observed in our updated review that included studies from 25 countries.

According to the included studies, CAM use is common amongst patients with diabetes for the purpose of diabetes management. Most of the studies showed that the participants they recruited used CAM as an additional approach to conventional treatment, while in other studies, the reason for CAM use (additional or alternative) was not specified. Only seven studies reported that some patients with diabetes used CAM as the sole means of managing their diabetes. Most of the included studies were conducted in healthcare settings. Therefore, patients who do not use conventional treatments for diabetes may not have been included. The prevalence of patients with diabetes who use CAM in the general population with diabetes is hence likely to be higher than the estimates provided by the included studies.

The meta-analysis of the prevalence data demonstrated extreme variation in prevalence of CAM use amongst patients with diabetes across studies.

### Strengths and limitations

This systematic review was a protocol driven review with a pre-specified aim, objectives and methodology. A range of relevant databases were used which covered the prevalence of CAM used by patients with diabetes globally. Data collection methods varied amongst studies. Some studies used structured questionnaires while other studies used semi-structured questionnaires. In addition there was a wide variation in the nature of the study settings. The content of the questionnaires used to collect the data on prevalence and nature of CAMs are likely to influence patient response. Therefore, the included studies may have underreported the nature and extent of CAM use by study participants. In addition, our study only included studies published in the English language.

### Implications for practice and research

This systematic review shows that CAM use amongst patients with diabetes is prevalent in many populations. This review suggests that healthcare professionals should consider use of CAM by patients with diabetes when advising them about using their prescribed treatments and monitor their medication adherence while using any forms of CAM. They should also be aware of patients’ use of herbal supplements as some forms of herbal medicine can lead to herb–drug interactions [57]. For example, the most frequently mentioned herbal supplement used by patients with diabetes was cinnamon. It is reported that cinnamon has a potentiating effect on diabetic drugs increasing the risk of hypoglycaemia [58]. For example, cinnamon shows an inhibitory effect on the CYP3A4 enzyme in rabbits which potentiates the effect of pioglitazone if combined with cinnamon, leading to a hypoglycaemic effect [59]. *Aloe vera*, which was the most frequently reported CAM by the studies included in our review, has been linked to potential interaction with 45 different drugs, including diabetic drugs such as glimepiride [60]. Concomitant use of *Aloe vera* and glimepiride can produce hypoglycaemic effects as *Aloe vera* has an inhibiting effect on ATP sensitive potassium channels in pancreatic β cells leading to additional release of insulin [61].

Understanding CAM use patterns and considering any possible interactions between them and other potential medications could help healthcare professionals to appropriately minimize drug-related problems or herb–drug interactions. It could help them to encourage their patient to discuss their CAM use and offer the opportunity to provide better advice for patients with diabetes.

The observed prevalence of CAM, and the many varieties of CAM that are used by patients with diabetes, call for revision of diabetes management guidelines. The National Institute for Health and Care Excellence (NICE) guideline on management of diabetes does not explicitly advise healthcare professionals to discuss patient use of herbal medicines or CAM in their consultation [6]. Guidelines should enable healthcare professionals to counsel patients with diabetes, their families and carers, who should all be educated about the safe use of CAM in conjunction with prescribed medicines.

Owing to the variable and often high prevalence of CAM use amongst patients with diabetes worldwide, research that generates evidence-based information about CAM is needed. This includes effectiveness and safety profiles of commonly used CAMs, including herbal medicines as identified in this systematic review.

This systematic review has identified that on average up to two-thirds of patients who use CAM do not disclose this to their healthcare professionals. Use of CAM such as herbal medicines could be incorporated as part of the comprehensive medication review services offered at community pharmacies and primary care [62].

Future studies need to consider the perspectives of patients with diabetes who do not visit conventional healthcare facilities for the management of diabetes to provide a better estimate of prevalence rates. In addition, there is a need to gather evidence on the factors that affect use and non-use of CAM by patients with diabetes.

## Conclusion

A wide variation in prevalence rate of CAM use in diabetes (8–89%) was observed, and pooled prevalence of CAM use was 51%. Our findings show that CAM use by patients with diabetes is common. Healthcare professionals should be aware of the use of CAM by patients with diabetes to ensure treatment optimization and medication adherence. Future studies should incorporate patient and healthcare professionals’ perspectives of CAM use in diabetes, evaluate patient outcomes through the use of healthcare databases and carefully designed prospective studies, and identify opportunities to promote rational use of CAM through evidence-based guidelines and patient-centred approaches.

## Supplementary Information

ESM 1 (DOCX 170 kb)

## Data Availability

All data in relation to this study are presented in this manuscript.
