# Supplementary material for: Global prevalence and types of complementary and alternative medicines use amongst adults with diabetes: systematic review and meta-analysis
Source: Eur J Clin Pharmacol. 2021 Mar 8;77(9):1259–74. doi: 10.1007/s00228-021-03097-x (PMC8346452; doi:10.1007/s00228-021-03097-x)
Supplement: Supplementary file 1 — (DOCX 170 kb) [file 228_2021_3097_MOESM1_ESM.docx]

Table S1: Search terms used for databases searches.

| 1. Acupressure 2. Acupuncture 3. Alternative 4. Aromatherapy 5. Ayurveda 6. Balneotherapy 7. Biofeedback 8. Complementary 9. Cupping 10. Functional medicine 11. Herbal 12. Homeopathy 13. Hypnotherapy 14. Leeching 15. Naturopathy 16. Oriental 17. Persian 18. Reflexology 19. Reiki 20. Traditional African Medicine 21. Traditional Arabic Medicine | 1. Traditional Chinese Medicine 2. Traditional Medicine 3. Traditional Persian Medicine 4. Yoga 5. 1 OR 2 OR 3 OR 4 OR 5 OR 6 OR 7 OR 8 OR 9 OR 10 OR 11 OR 12 OR 13 OR 14 OR 15 OR 16 OR 17 OR 18 OR 19 OR 20 OR 21 OR 22 OR 23 OR 23 OR 24 OR 25 6. Diabetes 7. Type 1 diabetes 8. Type 2 Diabetes 9. Pre-diabetes 10. Hyperglycaemia 11. Blood glucose 12. 27 OR 28 OR 29 OR 30 OR 31 OR 32 13. 26 AND 33 | 1. Among 2. Use 3. Prevalence 4. Beliefs 5. Attitude 6. Knowledge 7. Practice 8. Views 9. Behaviour 10. Proportion 11. Common 12. Survey 13. Interview 14. Frequent 15. Pattern 16. Trend 17. 35 OR 36 OR 37 OR 38 OR 39 OR 40 OR 41 OR 42 OR 43 OR 45 OR 46 OR 47 OR 48 OR 49 OR 50 18. 26 AND 33 AND 51 |
| --- | --- | --- |

Table S2: Critical Appraisal Summary using Joanna Briggs Institute Critical Appraisal tools (JBI) for quality assessment

| S | Author and date | Country | Study design | Yes | No | Unclear | Not applicable |
| --- | --- | --- | --- | --- | --- | --- | --- |
| 1 | Yildirim & Marakoglu, 2018 | Turkey | *CS* | 6 | 0 | 2 | 0 |
| 2 | Rhee, Westberg, & Harris, 2018 | USA | CS | 7 | 0 | 1 | 0 |
| 3 | Mekuria et al., 2018 | Ethiopia | CS | 7 | 1 | 0 | 0 |
| 4 | Karaman et al., 2018 | Turkey | CS | 6 | 1 | 0 | 1 |
| 5 | Candar et al., 2018 | Turkey | CS | 6 | 1 | 1 | 0 |
| 6 | Avci., 2018 | Turkey | CS | 6 | 1 | 1 | 0 |
| 7 | Andrews, Wyne, & Svenson, 2018 | Guatemala | CS | 5 | 2 | 1 | 0 |
| 8 | Amaeze et al., 2018 | Nigeria | CS | 5 | 2 | 1 | 0 |
| 9 | Mohamed Ali, & Mahfouz, 2014 | Sudan | CS | 5 | 2 | 1 | 0 |
| 10 | Vishnu, Mini & Thankappan, 2017 | India | CS | 5 | 2 | 1 | 0 |
| 11 | Putthapiban, et.al., 2017 | Thailand | CS | 6 | 1 | 1 | 0 |
| 12 | Kamel et al., 2017 | Saudi | CS | 4 | 3 | 1 | 0 |
| 13 | Ashur et al., 2017 | Libya | CS | 3 | 4 | 1 | 0 |
| 14 | Al-garni, Al-Raddadi & Al-Amri, 2017 | Saudi | CS | 5 | 2 | 1 | 0 |
| 15 | Wanchai & Phrompayak, 2016 | Thailand | CS | 5 | 2 | 1 | 0 |
| 16 | Lunyera et al., 2016 | Tanzania | CS | 5 | 2 | 1 | 0 |
| 17 | Bahroom, Shamsul & Rotina, 2016 | Malaysia | CS | 7 | 1 | 0 | 0 |
| 18 | Azizi-Fini, et. al, 2016 | Iran | CS | 7 | 1 | 0 | 0 |
| 19 | Al-Eidi et al., 2016 | Saudi | CS | 6 | 2 | 0 | 0 |
| 20 | Koren et al., 2015 | Israel | CS | 5 | 2 | 1 | 0 |
| 21 | Hashempur et al., 2015 | Iran | CS | 7 | 1 | 0 | 0 |
| 22 | Devi et al., 2015 | India | CS | 5 | 2 | 1 | 0 |
| 23 | Damnjanovic et al., 2015 | Serbia | CS | 5 | 2 | 1 | 0 |
| 24 | Alami et al., 2015 | Morocco | CS | 6 | 2 | 0 | 0 |
| 25 | Nguyen et al., 2014 | USA | CS | 6 | 2 | 0 | 0 |
| 26 | Naja et al., 2014 | Lebanon | CS | 7 | 1 | 0 | 0 |
| 27 | Medagama et al., 2014 | Sri Lanka | CS | 6 | 1 | 1 | 0 |
| 28 | Chao et al., 2014 | USA | CS | 4 | 3 | 1 | 0 |
| 29 | Khalil et al., 2013 | Egypt | CS | 3 | 2 | 3 | 0 |
| 30 | Fan et al., 2013 | Singapore | CS | 6 | 0 | 2 | 0 |
| 31 | Ching et al., 2013 | Malaysia | CS | 7 | 1 | 0 | 0 |
| 32 | Lui et al., 2012 | Australia | DC | 6 | 0 | 5 | 0 |
| 33 | Ali-Shtayehet et al., 2012 | Palestine | CS | 5 | 2 | 1 | 0 |
| 34 | Wazaify et al., 2011 | Jordan | CS | 6 | 2 | 0 | 0 |
| 35 | Sethi, Srivastava & Madhu, 2011 | India | CS | 5 | 2 | 1 | 0 |
| 36 | Fabian et al., 2011 | Austria | CS | 7 | 1 | 0 | 0 |
| 37 | Bradley et al., 2011 | USA | CS | 6 | 1 | 1 | 0 |
| 38 | Khalaf & Whitford, 2010 | Bahrain | CS | 5 | 1 | 2 | 0 |

CS= Cross-Sectional, DC= data obtained from cohort study.

Table S3: Herbal and dietary supplements cited in included studies and the frequency of citations (each out of 41 studies)

| \| 1 \| **Abeere** \| **1** \| \| --- \| --- \| --- \| \| 2 \| Abyssinian rose \| 1 \| \| 3 \| Agbo iba \| 1 \| \| 4 \| Agbo jedi \| 1 \| \| 5 \| Agunmu \| 1 \| \| 6 \| Ajenjo \| 1 \| \| 7 \| Ajuga iva \| 1 \| \| 8 \| Almonds \| 5 \| \| 9 \| Aloe vera \| 14 \| \| 10 \| Anise \| 5 \| \| 11 \| Apple \| 1 \| \| 12 \| Argan \| 1 \| \| 13 \| Artichoke \| 4 \| \| 14 \| Arugula \| 1 \| \| 15 \| Austrian oak \| 1 \| \| 16 \| Avocado leaves \| 2 \| \| 17 \| Barley \| 2 \| \| 18 \| Basil leaf \| 2 \| \| 19 \| Bay laurel \| 1 \| \| 20 \| Bayleaf \| 1 \| \| 21 \| Bean \| 1 \| \| 22 \| Beet \| 1 \| \| 23 \| Bitter aloe \| 1 \| \| 24 \| Bitter gourd \| 3 \| \| 25 \| Bi­tter leaf \| 1 \| \| 26 \| Bitter melon \| 6 \| \| 27 \| Black calla \| 1 \| \| 28 \| Black mulberry \| 2 \| \| 29 \| Black seed \| 12 \| \| 30 \| Blackberry \| 1 \| \| 31 \| Blueberry \| 4 \| \| 32 \| Breadnut \| 1 \| \| 33 \| Cabbage \| 2 \| \| 34 \| Camomile \| 2 \| \| 35 \| Candyleaf \| 1 \| \| 36 \| Caraway \| 1 \| \| 37 \| Cardamom \| 1 \| \| 38 \| Cardoon \| 1 \| \| 39 \| Celery \| 3 \| \| 40 \| Chamomile \| 3 \| \| 41 \| Charlock \| 1 \| \| 42 \| Cherry stalk \| 1 \| \| 43 \| Chichipin \| 1 \| \| 44 \| Chickpea \| 3 \| \| 45 \| Chicory \| 1 \| \| 46 \| Christ's thorn jujube \| 1 \| \| 47 \| Cinnamon \| 18 \| \| 48 \| Cloves \| 2 \| \| 49 \| Cocoa powder \| 1 \| \| 50 \| Coconut \| 2 \| \| 51 \| Coenzyme Q10 \| 2 \| \| 52 \| Colocynth \| 2 \| \| 53 \| Common motherwort \| 1 \| \| 54 \| Common rue \| 2 \| \| 55 \| Coriander \| 4 \| \| 56 \| Costus Indian \| 1 \| \| 57 \| Crab Apple \| 1 \| \| 58 \| Cranberry \| 1 \| \| 59 \| Crownvetch \| 2 \| \| 60 \| Cultivated Mushroom \| 1 \| \| 61 \| Cumin \| 4 \| \| 62 \| Dandelion \| 2 \| \| 63 \| Dates (bitter) \| 3 \| \| 64 \| Dill \| 2 \| \| 65 \| Dong quai \| 1 \| \| 66 \| Doum \| 1 \| \| 67 \| Dyer's madder \| 1 \| \| 68 \| East African rosewood \| 2 \| \| 69 \| Ecballium \| 1 \| \| 70 \| Echinops \| 1 \| \| 71 \| Efinrin \| 1 \| \| 72 \| Eggplant \| 1 \| \| 73 \| Eucalyptus \| 1 \| \| 74 \| Ewe laali \| 1 \| \| 75 \| Ewe ogbon \| 1 \| \| 76 \| Felty germander \| 3 \| \| 77 \| Fennel \| 2 \| \| 78 \| Fenugreek \| 18 \| \| 79 \| Fig \| 2 \| \| 80 \| Fish oil \| 3 \| \| 81 \| Flaxeed \| 4 \| \| 82 \| Garden Cress \| 2 \| \| 83 \| Garlic \| 17 \| \| 84 \| Gboko cleanser \| 1 \| \| 85 \| Gentian \| 1 \| \| 86 \| Ginger \| 11 \| \| 87 \| Gingko \| 1 \| \| 88 \| Ginseng \| 6 \| \| 89 \| Globularia \| 1 \| \| 90 \| Glucosamine \| 4 \| \| 91 \| Goko Cleanser \| 1 \| \| 92 \| Golden Cotula \| 1 \| \| 93 \| Grapefruit \| 1 \| \| 94 \| Green chiretta \| 2 \| \| 95 \| Green tea \| 10 \| \| 96 \| Guava leaf \| 3 \| \| 97 \| Guduchi \| 1 \| \| 98 \| Gum plant \| 1 \| \| 99 \| Gurmar \| 1 \| \| 100 \| Gymnena \| 1 \| \| 101 \| Harmal \| 2 \| \| 102 \| Hawthorn \| 1 \| \| 103 \| heart-leaved moonseed \| 1 \| \| 104 \| Helichrysum \| 1 \| \| 105 \| Helteet \| 1 \| \| 106 \| Hibiscus \| 3 \| \| 107 \| Hierba mora \| 1 \| \| 108 \| Honey \| 3 \| \| 109 \| Horse wood \| 2 \| \| 110 \| Indian gooseberry \| 1 \| \| 111 \| Indian screw tree \| 1 \| \| 112 \| Ivy gourd \| 1 \| | \| 113 \| **Jaadah or Shangoura** \| **1** \| \| --- \| --- \| --- \| \| 114 \| Jamun \| 2 \| \| 115 \| Java tea \| 2 \| \| 116 \| Jiaogulan \| 1 \| \| 117 \| Johnson grass \| 1 \| \| 118 \| Juniper \| 2 \| \| 119 \| Karela \| 1 \| \| 120 \| Khella \| 1 \| \| 121 \| Kiwi \| 1 \| \| 122 \| Launaea \| 1 \| \| 123 \| Lemon \| 3 \| \| 124 \| Lemon verbena \| 1 \| \| 125 \| Lemongrass \| 1 \| \| 126 \| Lentil \| 1 \| \| 127 \| Lentisco \| 1 \| \| 128 \| Lettuce \| 1 \| \| 129 \| licorice \| 3 \| \| 130 \| Lime \| 4 \| \| 131 \| Lingzhi mushroom \| 1 \| \| 132 \| Loquat leaves \| 3 \| \| 133 \| Lupine seeds \| 5 \| \| 134 \| Mahaleb cherry \| 1 \| \| 135 \| Malt \| 1 \| \| 136 \| Mango leaves \| 1 \| \| 137 \| Manzana rosa \| 1 \| \| 138 \| Marjoram \| 3 \| \| 139 \| Mediterranean wild thyme \| 1 \| \| 140 \| Melon \| 1 \| \| 141 \| Milk \| 1 \| \| 142 \| Milk thistle \| 1 \| \| 143 \| Milkvetch \| 1 \| \| 144 \| Mint \| 1 \| \| 145 \| Misai Kuching \| 1 \| \| 146 \| Mistletoe \| 1 \| \| 147 \| Moringa \| 5 \| \| 148 \| Mountain banana \| 1 \| \| 149 \| Musk willow sweat \| 1 \| \| 150 \| Muskmelon \| 1 \| \| 151 \| Mustard seed \| 1 \| \| 152 \| Myrrh \| 3 \| \| 153 \| Myrtle \| 1 \| \| 154 \| Neem leaves \| 4 \| \| 155 \| Nerium \| 1 \| \| 156 \| Níspero \| 1 \| \| 157 \| Noni \| 1 \| \| 158 \| Oka baba \| 1 \| \| 159 \| Okra seeds and flowers \| 2 \| \| 160 \| Olibanum \| 1 \| \| 161 \| Olive leaf \| 10 \| \| 162 \| Omega-3 \| 2 \| \| 163 \| Onion \| 8 \| \| 164 \| Orange (peel) \| 1 \| \| 165 \| Oregano (Syrian) \| 1 \| \| 166 \| Oroki \| 1 \| \| 167 \| Orris Root \| 1 \| \| 168 \| Parsley \| 2 \| \| 169 \| Passion flower \| 1 \| \| 170 \| Pennyroyal \| 1 \| \| 171 \| Pigeon pea \| 1 \| \| 172 \| Pomegranate \| 6 \| \| 173 \| Prickly pear \| 2 \| \| 174 \| Prostrate speedwell \| 1 \| \| 175 \| Psyllium \| 1 \| \| 176 \| Psyllium \| 1 \| \| 177 \| Purslane \| 1 \| \| 178 \| Quince leaves \| 2 \| \| 179 \| Redcurrant \| 1 \| \| 180 \| Resveratrol \| 1 \| \| 181 \| Rock cherry \| 1 \| \| 182 \| Roman nettle \| 1 \| \| 183 \| Rose oil \| 1 \| \| 184 \| Rosehip \| 1 \| \| 185 \| Roselle \| 2 \| \| 186 \| Rosemary \| 4 \| \| 187 \| Ryeroot \| 1 \| \| 188 \| Sabah snake grass \| 1 \| \| 189 \| Sage \| 8 \| \| 190 \| Salacia reticulata \| 1 \| \| 191 \| Sarrasine \| 1 \| \| 192 \| Schenkia \| 1 \| \| 193 \| Senna \| 1 \| \| 194 \| Sesame \| 2 \| \| 195 \| Siberian aronia \| 1 \| \| 196 \| Soursop \| 1 \| \| 197 \| Soy \| 1 \| \| 198 \| Spiderwort \| 1 \| \| 199 \| Spineless yucca \| 1 \| \| 200 \| Splender \| 1 \| \| 201 \| St. John's wort \| 1 \| \| 202 \| Stinging nettle \| 6 \| \| 203 \| Swedish bitt­er \| 1 \| \| 204 \| Tamarisk \| 1 \| \| 205 \| Tasmanian blue gum \| 1 \| \| 206 \| Tetraclinis \| 1 \| \| 207 \| Thunbergia laurifolia \| 1 \| \| 208 \| Thyme \| 7 \| \| 209 \| Thymelaea \| 1 \| \| 210 \| Timboque \| 1 \| \| 211 \| Tres puntos \| 1 \| \| 212 \| Turmeric \| 2 \| \| 213 \| Verbena \| 1 \| \| 214 \| Verjuice \| 1 \| \| 215 \| Vitamins and minerals \| 8 \| \| 216 \| Walnut \| 4 \| \| 217 \| Watermelon \| 1 \| \| 218 \| Wheat \| 3 \| \| 219 \| White horehound \| 2 \| \| 220 \| Wormwood \| 5 \| \| 221 \| Yoyo Bi­tters \| 1 \| \| 222 \| Za'atar \| 2 \| \| 223 \| Ziziphus \| 4 \| |
| --- | --- | --- | --- | --- | --- | --- | --- | --- | --- | --- | --- | --- | --- | --- | --- | --- | --- | --- | --- | --- | --- | --- | --- | --- | --- | --- | --- | --- | --- | --- | --- | --- | --- | --- | --- | --- | --- | --- | --- | --- | --- | --- | --- | --- | --- | --- | --- | --- | --- | --- | --- | --- | --- | --- | --- | --- | --- | --- | --- | --- | --- | --- | --- | --- | --- | --- | --- | --- | --- | --- | --- | --- | --- | --- | --- | --- | --- | --- | --- | --- | --- | --- | --- | --- | --- | --- | --- | --- | --- | --- | --- | --- | --- | --- | --- | --- | --- | --- | --- | --- | --- | --- | --- | --- | --- | --- | --- | --- | --- | --- | --- | --- | --- | --- | --- | --- | --- | --- | --- | --- | --- | --- | --- | --- | --- | --- | --- | --- | --- | --- | --- | --- | --- | --- | --- | --- | --- | --- | --- | --- | --- | --- | --- | --- | --- | --- | --- | --- | --- | --- | --- | --- | --- | --- | --- | --- | --- | --- | --- | --- | --- | --- | --- | --- | --- | --- | --- | --- | --- | --- | --- | --- | --- | --- | --- | --- | --- | --- | --- | --- | --- | --- | --- | --- | --- | --- | --- | --- | --- | --- | --- | --- | --- | --- | --- | --- | --- | --- | --- | --- | --- | --- | --- | --- | --- | --- | --- | --- | --- | --- | --- | --- | --- | --- | --- | --- | --- | --- | --- | --- | --- | --- | --- | --- | --- | --- | --- | --- | --- | --- | --- | --- | --- | --- | --- | --- | --- | --- | --- | --- | --- | --- | --- | --- | --- | --- | --- | --- | --- | --- | --- | --- | --- | --- | --- | --- | --- | --- | --- | --- | --- | --- | --- | --- | --- | --- | --- | --- | --- | --- | --- | --- | --- | --- | --- | --- | --- | --- | --- | --- | --- | --- | --- | --- | --- | --- | --- | --- | --- | --- | --- | --- | --- | --- | --- | --- | --- | --- | --- | --- | --- | --- | --- | --- | --- | --- | --- | --- | --- | --- | --- | --- | --- | --- | --- | --- | --- | --- | --- | --- | --- | --- | --- | --- | --- | --- | --- | --- | --- | --- | --- | --- | --- | --- | --- | --- | --- | --- | --- | --- | --- | --- | --- | --- | --- | --- | --- | --- | --- | --- | --- | --- | --- | --- | --- | --- | --- | --- | --- | --- | --- | --- | --- | --- | --- | --- | --- | --- | --- | --- | --- | --- | --- | --- | --- | --- | --- | --- | --- | --- | --- | --- | --- | --- | --- | --- | --- | --- | --- | --- | --- | --- | --- | --- | --- | --- | --- | --- | --- | --- | --- | --- | --- | --- | --- | --- | --- | --- | --- | --- | --- | --- | --- | --- | --- | --- | --- | --- | --- | --- | --- | --- | --- | --- | --- | --- | --- | --- | --- | --- | --- | --- | --- | --- | --- | --- | --- | --- | --- | --- | --- | --- | --- | --- | --- | --- | --- | --- | --- | --- | --- | --- | --- | --- | --- | --- | --- | --- | --- | --- | --- | --- | --- | --- | --- | --- | --- | --- | --- | --- | --- | --- | --- | --- | --- | --- | --- | --- | --- | --- | --- | --- | --- | --- | --- | --- | --- | --- | --- | --- | --- | --- | --- | --- | --- | --- | --- | --- | --- | --- | --- | --- | --- | --- | --- | --- | --- | --- | --- | --- | --- | --- | --- | --- | --- | --- | --- | --- | --- | --- | --- | --- | --- | --- | --- | --- | --- | --- | --- | --- | --- | --- | --- | --- | --- | --- | --- | --- | --- | --- | --- | --- | --- | --- | --- | --- | --- | --- | --- | --- | --- | --- | --- | --- | --- | --- | --- | --- | --- | --- | --- | --- | --- | --- | --- | --- | --- | --- | --- | --- | --- | --- | --- | --- | --- | --- | --- | --- | --- | --- | --- | --- | --- | --- | --- | --- | --- | --- | --- | --- | --- | --- | --- | --- | --- | --- | --- | --- | --- | --- | --- | --- | --- | --- | --- | --- | --- | --- | --- | --- | --- | --- | --- | --- | --- | --- | --- | --- | --- | --- | --- | --- | --- | --- | --- | --- | --- | --- | --- | --- | --- | --- | --- | --- | --- | --- | --- | --- | --- | --- | --- | --- | --- | --- | --- | --- | --- | --- | --- | --- | --- | --- | --- | --- | --- | --- | --- | --- | --- | --- | --- | --- | --- | --- | --- | --- | --- | --- | --- | --- |

Figure S2: Prevelance ratio of CAM use between patients who have diabetes for more than 5 years verses patients who have diabetes for less than 5 years.

Figure S1: Prevelance ratio of CAM use between patients with no diabetic complications verses patients with diabetic complications.

CAM: Complementary and Alternative Medicine

Figure S4: Prevalence of CAM use in T2DM patients and T1DM patients

Figure S3 : Prevelance ratio of CAM use between male patients versus female patients.

.

CAM: Complementary and Alternative Medicine CAM: Complementary and Alternative Medicine

CAM: Complementary and Alternative Medicine; T2DM: Type 2 Diabetes Mellitus; T1DM: Type 1 Diabetes Mellitus

Figure S6 : Use of CAM as alternative treatment

Figure S5: Use of CAM as additional treatment

CAM: Complementary and Alternative Medicine CAM: Complementary and Alternative Medicine
